# (2,3-Di-2-pyridyl­pyrazine-κ^2^ *N* ^2^,*N* ^3^)diiodidoplatinum(II)

**DOI:** 10.1107/S1600536812023501

**Published:** 2012-05-31

**Authors:** Kwang Ha

**Affiliations:** a School of Applied Chemical Engineering, The Research Institute of Catalysis, Chonnam National University, Gwangju 500-757, Republic of Korea

## Abstract

The Pt^II^ ion in the title complex, [PtI_2_(C_14_H_10_N_4_)], exists in a distorted square-planar environment defined by the two pyridine N atoms of the chelating 2,3-di-2-pyridyl­pyrazine ligand and two iodide anions. The pyridine rings are inclined to the least-squares plane of the PtI_2_N_2_ unit [maximum deviation = 0.070 (3) Å] at 66.1 (2) and 65.9 (2)°; the pyrazine ring is perpendicular to this plane [dihedral angle = 89.7 (2)°]. Two inter­molecular C—H⋯I hydrogen bonds, both involving the same I atom as hydrogen-bond acceptor, generate a layer structure extending parallel to (001). Mol­ecules are stacked in columns along the *a* axis. Along the *b* axis, successive mol­ecules stack in opposite directions.

## Related literature

For [PtBr_2_(dpp)] and [PdI_2_(dpp)] (dpp = 2,3-di-2-pyridyl­pyrazine), see: Ha (2011*a* ▶,*b* ▶).
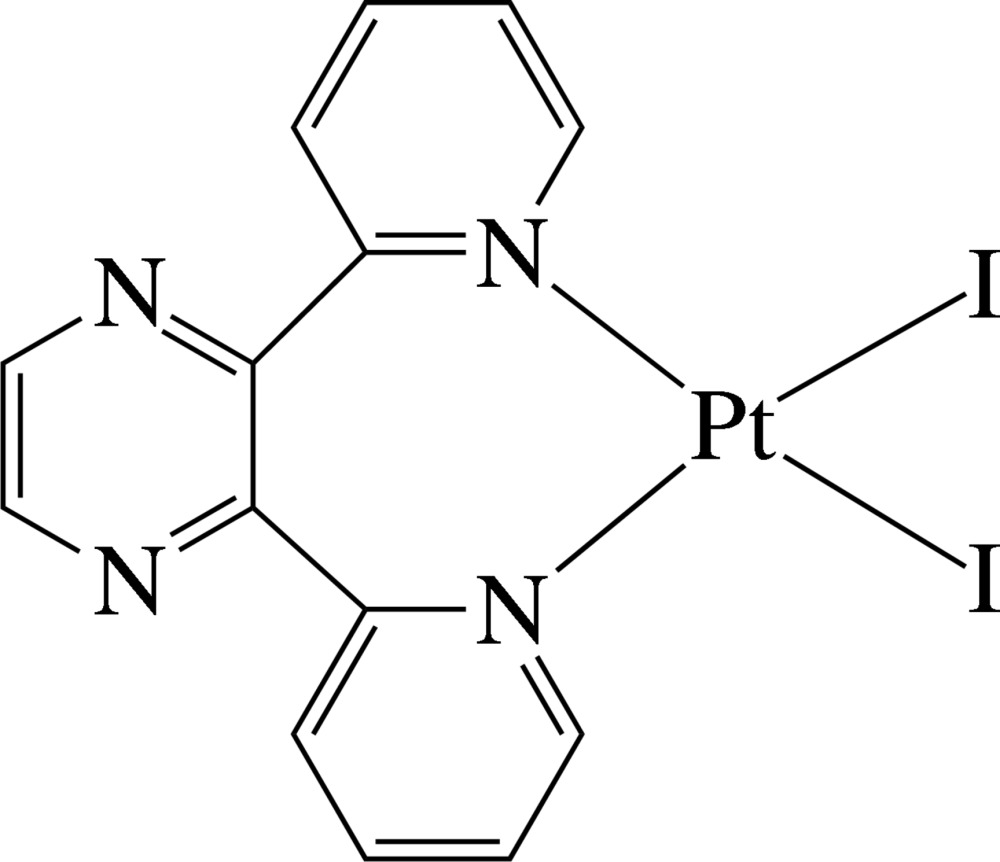


## Experimental

### 

#### Crystal data

[PtI_2_(C_14_H_10_N_4_)]
*M* *_r_* = 683.15
Monoclinic, 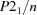

*a* = 8.7600 (7) Å
*b* = 15.4750 (12) Å
*c* = 12.5004 (10) Å
β = 102.660 (2)°
*V* = 1653.4 (2) Å^3^
*Z* = 4
Mo *K*α radiation
μ = 12.22 mm^−1^
*T* = 200 K
0.26 × 0.23 × 0.19 mm

#### Data collection

Bruker SMART 1000 CCD diffractometer
Absorption correction: multi-scan (*SADABS*; Bruker, 2000 ▶) *T*_min_ = 0.551, *T*_max_ = 1.000
10058 measured reflections
3215 independent reflections
2807 reflections with *I* > 2σ(*I*)
*R*_int_ = 0.034

#### Refinement

*R*[*F* ^2^ > 2σ(*F* ^2^)] = 0.029
*wR*(*F* ^2^) = 0.077
*S* = 1.07
3215 reflections
190 parameters
H-atom parameters constrained
Δρ_max_ = 1.30 e Å^−3^
Δρ_min_ = −1.26 e Å^−3^

### 

Data collection: *SMART* (Bruker, 2000 ▶); cell refinement: *SAINT* (Bruker, 2000 ▶); data reduction: *SAINT*; program(s) used to solve structure: *SHELXS97* (Sheldrick, 2008 ▶); program(s) used to refine structure: *SHELXL97* (Sheldrick, 2008 ▶); molecular graphics: *ORTEP-3* (Farrugia, 1997 ▶) and *PLATON* (Spek, 2009 ▶); software used to prepare material for publication: *SHELXL97*.

## Supplementary Material

Crystal structure: contains datablock(s) global, I. DOI: 10.1107/S1600536812023501/ng5273sup1.cif

Structure factors: contains datablock(s) I. DOI: 10.1107/S1600536812023501/ng5273Isup2.hkl

Additional supplementary materials:  crystallographic information; 3D view; checkCIF report

## Figures and Tables

**Table:** 

Pt1—N4	2.030 (6)
Pt1—N3	2.036 (5)
Pt1—I1	2.5805 (6)
Pt1—I2	2.5930 (6)

**Table:** 

N4—Pt1—N3	87.2 (2)
I1—Pt1—I2	93.14 (2)

**Table 2:** Hydrogen-bond geometry (Å, °)

*D*—H⋯*A*	*D*—H	H⋯*A*	*D*⋯*A*	*D*—H⋯*A*
C6—H6⋯I1^i^	0.95	3.04	3.694 (7)	127
C11—H11⋯I1^ii^	0.95	3.01	3.813 (8)	143

Symmetry codes: (i) 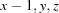; (ii) 
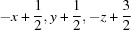
.

**Fig. 1.**
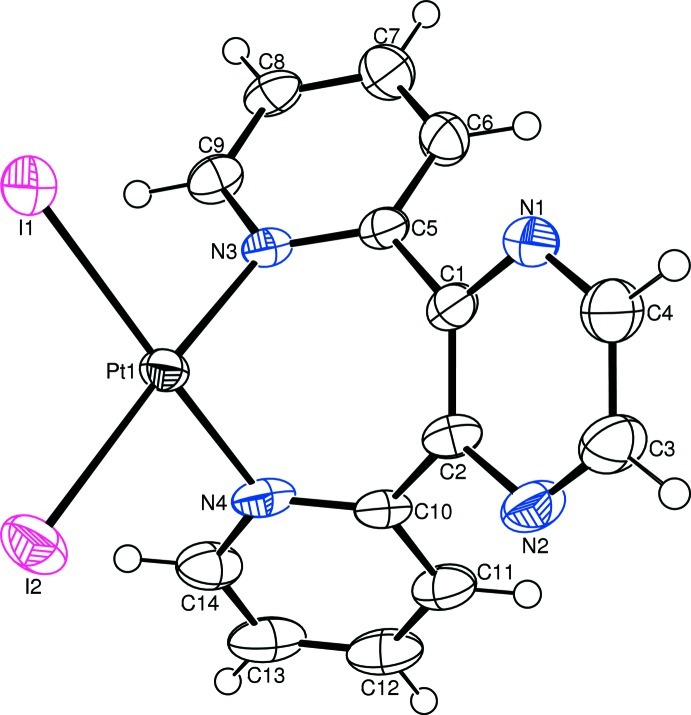
The molecular structure of the title complex, with atom numbering. Displacement ellipsoids are drawn at the 50% probability level for non-H atoms.

**Fig. 2.**
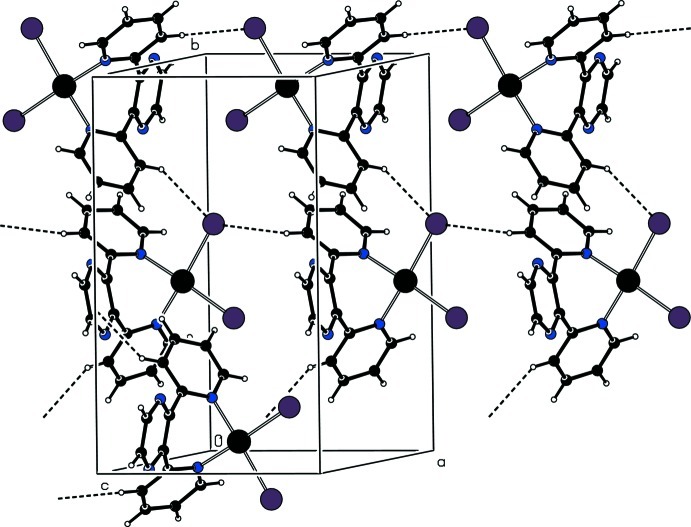
View of the hydrogen-bond interactions of the title complex. Hydrogen-bonds are drawn with dashed lines.

## supplementary crystallographic information

### Comment

The title complex, [PtI_2_(dpp)] (dpp = 2,3-di-2-pyridylpyrazine, C_14_H_10_N_4_), is a structural isomer of the previously reported Pt^II^ and Pd^II^ complexes, [PtBr_2_(dpp)] and [PdI_2_(dpp)] (Ha, 2011*a*,*b*).

The Pt^II^ ion has a slightly distorted square-planar environment defined by the two pyridine N atoms of the chelating dpp ligand and two iodide anions (Fig. 1). The N3—Pt1—N4 chelate angle of 87.2 (2)° and I—I repelling contribute the distortion of square, and therefore the *trans* axes are slightly bent [<I1—Pt1—N4 = 174.46 (16)° and <I2—Pt1—N3 = 176.99 (15)°]. The Pt—N and Pt—I bond lengths are nearly equivalent, respectively (Table 1). In the crystal, the two pyridine rings are considerably inclined to the least-squares plane of the PtI_2_N_2_ unit [maximum deviation = 0.070 (3) Å], with dihedral angles of 66.1 (2)° and 65.9 (2)°. The nearly planar pyrazine ring [maximum deviation = 0.014 (5) Å] is perpendicular to the unit plane, with a dihedral angle of 89.7 (2)°. The dihedral angle between the two pyridine rings is 80.0 (2)°. Two independent weak intermolecular C—H···I hydrogen bonds, both involving the same I atom as a hydrogen-bond acceptor, give rise to chains running along the a and b axes, generating a layer structure extending parallel to the *ab* plane (Fig. 2 and Table 2). The complexes are stacked in columns along the *a* axis. When viewed down the *b* axis, the successive complexes stack in opposite directions. In the columns, numerous inter- and intramolecular π-π interactions between the six-membered rings are present, the shortest ring centroid-centroid distance being 3.935 (4) Å.

### Experimental

The title complex was obtained as a byproduct from the reaction of K_2_PtCl_4_ (0.2089 g, 0.503 mmol) with 2,3-di-2-pyridylpyrazine (0.1198 g, 0.511 mmol) and KI (0.6785 g, 4.087 mmol) in H_2_O (20 ml)/MeOH (30 ml). The reaction mixture was stirred for 6 h at room temperature; the precipitate that formd was separated by filtration, washed with H_2_O and MeOH, and then collected to give the main product as a reddish brown powder (0.2638 g). The yellow by-product (0.0388 g) was obtained from the mixture of filtrate and washing solution. Crystals were obtained by slow evaporation from a CH_3_NO_2_ solution of the by-product.

### Refinement

H atoms were included in calculated positions and treated as riding atoms: C—H = 0.95 Å with *U*_iso_(H) = 1.2*U*_eq_(C). The highest peak (1.30 e Å^-3^) and the deepest hole (-1.26 e Å^-3^) in the difference Fourier map are located 0.26 Å and 0.63 Å, respectively, from the atoms Pt1 and I1.

### Crystal data

**Table:**

[PtI_2_(C_14_H_10_N_4_)]	*F*(000) = 1224
*M**_r_* = 683.15	*D*_x_ = 2.744 Mg m^−^^3^
Monoclinic, *P*2_1_/*n*	Mo *K*α radiation, λ = 0.71073 Å
Hall symbol: -P 2yn	Cell parameters from 6167 reflections
*a* = 8.7600 (7) Å	θ = 2.6–26.0°
*b* = 15.4750 (12) Å	µ = 12.22 mm^−^^1^
*c* = 12.5004 (10) Å	*T* = 200 K
β = 102.660 (2)°	Block, yellow
*V* = 1653.4 (2) Å^3^	0.26 × 0.23 × 0.19 mm
*Z* = 4	

### Data collection

**Table:**

Bruker SMART 1000 CCD diffractometer	3215 independent reflections
Radiation source: fine-focus sealed tube	2807 reflections with *I* > 2σ(*I*)
Graphite monochromator	*R*_int_ = 0.034
φ and ω scans	θ_max_ = 26.0°, θ_min_ = 2.1°
Absorption correction: multi-scan (*SADABS*; Bruker, 2000)	*h* = −10→10
*T*_min_ = 0.551, *T*_max_ = 1.000	*k* = −19→13
10058 measured reflections	*l* = −15→15

### Refinement

**Table:**

Refinement on *F*^2^	Primary atom site location: structure-invariant direct methods
Least-squares matrix: full	Secondary atom site location: difference Fourier map
*R*[*F*^2^ > 2σ(*F*^2^)] = 0.029	Hydrogen site location: inferred from neighbouring sites
*wR*(*F*^2^) = 0.077	H-atom parameters constrained
*S* = 1.07	*w* = 1/[σ^2^(*F*_o_^2^) + (0.0341*P*)^2^ + 4.3824*P*] where *P* = (*F*_o_^2^ + 2*F*_c_^2^)/3
3215 reflections	(Δ/σ)_max_ = 0.001
190 parameters	Δρ_max_ = 1.30 e Å^−^^3^
0 restraints	Δρ_min_ = −1.26 e Å^−^^3^

### Special details

**Table:**

Geometry. All e.s.d.'s (except the e.s.d. in the dihedral angle between two l.s. planes) are estimated using the full covariance matrix. The cell e.s.d.'s are taken into account individually in the estimation of e.s.d.'s in distances, angles and torsion angles; correlations between e.s.d.'s in cell parameters are only used when they are defined by crystal symmetry. An approximate (isotropic) treatment of cell e.s.d.'s is used for estimating e.s.d.'s involving l.s. planes.
Refinement. Refinement of *F*^2^ against ALL reflections. The weighted *R*-factor *wR* and goodness of fit *S* are based on *F*^2^, conventional *R*-factors *R* are based on *F*, with *F* set to zero for negative *F*^2^. The threshold expression of *F*^2^ > σ(*F*^2^) is used only for calculating *R*-factors(gt) *etc*. and is not relevant to the choice of reflections for refinement. *R*-factors based on *F*^2^ are statistically about twice as large as those based on *F*, and *R*- factors based on ALL data will be even larger.

### Fractional atomic coordinates and isotropic or equivalent isotropic displacement parameters (Å^2^)

**Table:**

	*x*	*y*	*z*	*U*_iso_*/*U*_eq_	
Pt1	0.47513 (3)	0.062468 (17)	0.68112 (2)	0.02787 (10)	
I1	0.62382 (6)	−0.08242 (3)	0.69367 (4)	0.04126 (14)	
I2	0.73035 (6)	0.15223 (4)	0.73544 (4)	0.04772 (16)	
N1	0.1840 (7)	0.0062 (4)	0.9040 (5)	0.0373 (14)	
N2	0.2411 (8)	0.1831 (4)	0.9096 (5)	0.0425 (15)	
N3	0.2701 (6)	−0.0044 (3)	0.6444 (4)	0.0280 (12)	
N4	0.3445 (7)	0.1720 (4)	0.6578 (5)	0.0369 (14)	
C1	0.2034 (8)	0.0481 (4)	0.8137 (6)	0.0308 (15)	
C2	0.2337 (8)	0.1370 (4)	0.8164 (6)	0.0331 (15)	
C3	0.2248 (10)	0.1399 (5)	0.9984 (6)	0.047 (2)	
H3	0.2330	0.1703	1.0654	0.056*	
C4	0.1965 (10)	0.0532 (5)	0.9959 (6)	0.047 (2)	
H4	0.1853	0.0251	1.0613	0.057*	
C5	0.1726 (7)	−0.0069 (4)	0.7139 (5)	0.0286 (14)	
C6	0.0443 (8)	−0.0605 (5)	0.6955 (6)	0.0375 (17)	
H6	−0.0224	−0.0617	0.7460	0.045*	
C7	0.0125 (8)	−0.1121 (5)	0.6041 (7)	0.0444 (19)	
H7	−0.0752	−0.1498	0.5913	0.053*	
C8	0.1095 (8)	−0.1086 (5)	0.5310 (6)	0.0383 (17)	
H8	0.0890	−0.1434	0.4668	0.046*	
C9	0.2355 (9)	−0.0544 (5)	0.5521 (6)	0.0343 (16)	
H9	0.3012	−0.0513	0.5011	0.041*	
C10	0.2419 (8)	0.1915 (4)	0.7201 (6)	0.0322 (15)	
C11	0.1480 (9)	0.2633 (5)	0.6997 (7)	0.0432 (19)	
H11	0.0769	0.2761	0.7450	0.052*	
C12	0.1573 (10)	0.3169 (5)	0.6127 (7)	0.052 (2)	
H12	0.0925	0.3666	0.5970	0.062*	
C13	0.2640 (11)	0.2964 (5)	0.5491 (6)	0.053 (2)	
H13	0.2735	0.3322	0.4891	0.064*	
C14	0.3539 (10)	0.2253 (5)	0.5731 (6)	0.0449 (19)	
H14	0.4265	0.2120	0.5291	0.054*	

### Atomic displacement parameters (Å^2^)

**Table:**

	*U*^11^	*U*^22^	*U*^33^	*U*^12^	*U*^13^	*U*^23^
Pt1	0.03372 (16)	0.02391 (16)	0.02724 (15)	−0.00575 (11)	0.00941 (11)	−0.00250 (10)
I1	0.0362 (3)	0.0397 (3)	0.0496 (3)	0.0012 (2)	0.0132 (2)	0.0003 (2)
I2	0.0505 (3)	0.0517 (3)	0.0432 (3)	−0.0247 (3)	0.0152 (2)	−0.0113 (2)
N1	0.046 (3)	0.029 (3)	0.040 (3)	−0.003 (3)	0.016 (3)	0.000 (3)
N2	0.061 (4)	0.030 (3)	0.037 (3)	0.000 (3)	0.012 (3)	−0.008 (3)
N3	0.035 (3)	0.019 (3)	0.030 (3)	0.000 (2)	0.006 (2)	−0.001 (2)
N4	0.055 (4)	0.020 (3)	0.034 (3)	−0.004 (3)	0.005 (3)	−0.004 (2)
C1	0.031 (4)	0.026 (4)	0.035 (4)	0.002 (3)	0.007 (3)	−0.005 (3)
C2	0.039 (4)	0.024 (4)	0.034 (4)	−0.003 (3)	0.002 (3)	−0.006 (3)
C3	0.065 (5)	0.039 (5)	0.036 (4)	−0.005 (4)	0.012 (4)	−0.010 (4)
C4	0.066 (5)	0.045 (5)	0.034 (4)	−0.003 (4)	0.019 (4)	−0.002 (4)
C5	0.031 (3)	0.021 (3)	0.033 (3)	0.002 (3)	0.006 (3)	−0.001 (3)
C6	0.032 (4)	0.039 (4)	0.042 (4)	−0.005 (3)	0.011 (3)	−0.006 (3)
C7	0.030 (4)	0.044 (5)	0.058 (5)	−0.008 (4)	0.007 (3)	−0.006 (4)
C8	0.042 (4)	0.028 (4)	0.041 (4)	−0.001 (3)	0.002 (3)	−0.012 (3)
C9	0.043 (4)	0.032 (4)	0.025 (3)	0.001 (3)	0.002 (3)	−0.006 (3)
C10	0.041 (4)	0.019 (3)	0.034 (4)	−0.004 (3)	0.005 (3)	−0.002 (3)
C11	0.046 (4)	0.025 (4)	0.055 (5)	0.005 (3)	0.004 (4)	0.000 (3)
C12	0.060 (5)	0.025 (4)	0.060 (5)	0.004 (4)	−0.007 (4)	0.006 (4)
C13	0.090 (7)	0.029 (4)	0.035 (4)	−0.008 (5)	0.002 (4)	0.002 (4)
C14	0.071 (5)	0.032 (4)	0.032 (4)	−0.007 (4)	0.012 (4)	0.003 (3)

### Geometric parameters (Å, º)

**Table:**

Pt1—N4	2.030 (6)	C4—H4	0.9500
Pt1—N3	2.036 (5)	C5—C6	1.375 (10)
Pt1—I1	2.5805 (6)	C6—C7	1.372 (11)
Pt1—I2	2.5930 (6)	C6—H6	0.9500
N1—C4	1.343 (10)	C7—C8	1.379 (11)
N1—C1	1.345 (9)	C7—H7	0.9500
N2—C3	1.329 (10)	C8—C9	1.366 (10)
N2—C2	1.355 (9)	C8—H8	0.9500
N3—C5	1.346 (8)	C9—H9	0.9500
N3—C9	1.366 (8)	C10—C11	1.373 (10)
N4—C10	1.346 (9)	C11—C12	1.384 (11)
N4—C14	1.360 (9)	C11—H11	0.9500
C1—C2	1.400 (10)	C12—C13	1.391 (13)
C1—C5	1.486 (9)	C12—H12	0.9500
C2—C10	1.485 (10)	C13—C14	1.347 (12)
C3—C4	1.364 (11)	C13—H13	0.9500
C3—H3	0.9500	C14—H14	0.9500
			
N4—Pt1—N3	87.2 (2)	C6—C5—C1	118.5 (6)
N4—Pt1—I1	174.46 (16)	C7—C6—C5	120.0 (7)
N3—Pt1—I1	88.89 (15)	C7—C6—H6	120.0
N4—Pt1—I2	90.97 (17)	C5—C6—H6	120.0
N3—Pt1—I2	176.99 (15)	C6—C7—C8	119.2 (7)
I1—Pt1—I2	93.14 (2)	C6—C7—H7	120.4
C4—N1—C1	117.0 (6)	C8—C7—H7	120.4
C3—N2—C2	117.3 (6)	C9—C8—C7	119.0 (7)
C5—N3—C9	118.3 (6)	C9—C8—H8	120.5
C5—N3—Pt1	121.5 (4)	C7—C8—H8	120.5
C9—N3—Pt1	120.0 (5)	N3—C9—C8	122.1 (7)
C10—N4—C14	118.5 (6)	N3—C9—H9	118.9
C10—N4—Pt1	122.1 (5)	C8—C9—H9	118.9
C14—N4—Pt1	119.4 (5)	N4—C10—C11	121.5 (7)
N1—C1—C2	120.8 (6)	N4—C10—C2	119.9 (6)
N1—C1—C5	113.3 (6)	C11—C10—C2	118.5 (7)
C2—C1—C5	125.6 (6)	C10—C11—C12	119.7 (8)
N2—C2—C1	120.7 (7)	C10—C11—H11	120.2
N2—C2—C10	113.4 (6)	C12—C11—H11	120.2
C1—C2—C10	125.5 (6)	C11—C12—C13	118.3 (8)
N2—C3—C4	121.9 (7)	C11—C12—H12	120.8
N2—C3—H3	119.1	C13—C12—H12	120.8
C4—C3—H3	119.1	C14—C13—C12	119.5 (8)
N1—C4—C3	122.2 (7)	C14—C13—H13	120.2
N1—C4—H4	118.9	C12—C13—H13	120.2
C3—C4—H4	118.9	C13—C14—N4	122.5 (8)
N3—C5—C6	121.4 (6)	C13—C14—H14	118.8
N3—C5—C1	120.1 (6)	N4—C14—H14	118.8
			
N4—Pt1—N3—C5	−71.2 (5)	N1—C1—C5—C6	43.3 (9)
I1—Pt1—N3—C5	112.7 (5)	C2—C1—C5—C6	−130.3 (8)
N4—Pt1—N3—C9	114.8 (5)	N3—C5—C6—C7	−0.7 (11)
I1—Pt1—N3—C9	−61.4 (5)	C1—C5—C6—C7	180.0 (7)
N3—Pt1—N4—C10	62.0 (5)	C5—C6—C7—C8	−0.8 (12)
I2—Pt1—N4—C10	−115.6 (5)	C6—C7—C8—C9	0.6 (12)
N3—Pt1—N4—C14	−114.3 (6)	C5—N3—C9—C8	−2.5 (10)
I2—Pt1—N4—C14	68.1 (5)	Pt1—N3—C9—C8	171.7 (5)
C4—N1—C1—C2	−0.5 (10)	C7—C8—C9—N3	1.1 (11)
C4—N1—C1—C5	−174.5 (6)	C14—N4—C10—C11	0.0 (10)
C3—N2—C2—C1	2.6 (11)	Pt1—N4—C10—C11	−176.3 (5)
C3—N2—C2—C10	175.9 (7)	C14—N4—C10—C2	−177.2 (6)
N1—C1—C2—N2	−1.3 (11)	Pt1—N4—C10—C2	6.5 (8)
C5—C1—C2—N2	171.8 (7)	N2—C2—C10—N4	129.3 (7)
N1—C1—C2—C10	−173.8 (7)	C1—C2—C10—N4	−57.7 (10)
C5—C1—C2—C10	−0.6 (11)	N2—C2—C10—C11	−47.9 (9)
C2—N2—C3—C4	−2.1 (12)	C1—C2—C10—C11	125.1 (8)
C1—N1—C4—C3	1.1 (12)	N4—C10—C11—C12	0.5 (11)
N2—C3—C4—N1	0.2 (14)	C2—C10—C11—C12	177.7 (7)
C9—N3—C5—C6	2.3 (9)	C10—C11—C12—C13	−0.6 (12)
Pt1—N3—C5—C6	−171.8 (5)	C11—C12—C13—C14	0.3 (12)
C9—N3—C5—C1	−178.3 (6)	C12—C13—C14—N4	0.1 (12)
Pt1—N3—C5—C1	7.5 (8)	C10—N4—C14—C13	−0.3 (11)
N1—C1—C5—N3	−136.1 (6)	Pt1—N4—C14—C13	176.1 (6)
C2—C1—C5—N3	50.3 (10)		

### Hydrogen-bond geometry (Å, º)

**Table:**

*D*—H···*A*	*D*—H	H···*A*	*D*···*A*	*D*—H···*A*
C6—H6···I1^i^	0.95	3.04	3.694 (7)	127
C11—H11···I1^ii^	0.95	3.01	3.813 (8)	143

Symmetry codes: (i) *x*−1, *y*, *z*; (ii) −*x*+1/2, *y*+1/2, −*z*+3/2.
